# Simvastatin Sensitizes Radioresistant Prostate Cancer Cells by Compromising DNA Double-Strand Break Repair

**DOI:** 10.3389/fphar.2018.00600

**Published:** 2018-06-13

**Authors:** Yu-An Chen, Hua-Wei Shih, Yi-Chun Lin, Hui-Ying Hsu, Tsu-Fang Wu, Chen-Han Tsai, Chia-Lin Wu, Hui-Yu Wu, Jer-Tsong Hsieh, Chih-Hsin Tang, Chih-Ho Lai

**Affiliations:** ^1^Graduate Institute of Basic Medical Science, School of Medicine, China Medical University, Taichung, Taiwan; ^2^Department of Applied Cosmetology, Hung Kuang University, Taichung, Taiwan; ^3^Department of Microbiology and Immunology, Graduate Institute of Biomedical Sciences, College of Medicine, Chang Gung University, Taoyuan, Taiwan; ^4^Department of Biochemistry, College of Medicine, Chang Gung University, Taoyuan, Taiwan; ^5^Molecular Infectious Disease Research Center, Department of Neurology, Chang Gung Memorial Hospital, Linkou, Taiwan; ^6^Department of Urology, University of Texas Southwestern Medical Center, Dallas, TX, United States; ^7^Graduate Institute of Biomedical Sciences, School of Medicine, China Medical University, Taichung, Taiwan; ^8^Department of Nursing, Asia University, Taichung, Taiwan

**Keywords:** cholesterol, HMG-CoA reductase, simvastatin, prostate cancer, radioresistance, DNA double-strand break

## Abstract

Prostate cancer (PCa) is one of the most prevalent male cancers in western world. Radiation therapy (RT) is commonly used to treat PCa patients. However, a certain proportion of patients develop radioresistant PCa cells, which results in metastatic disease. Statins, which inhibit 3-hydroxy-3-methyl glutaryl coenzyme A (HMG-CoA) reductase, are commonly used to treat hypercholesterolemia, exhibiting beneficial effects on cardiovascular diseases and on several types of cancers, including PCa. However, the mechanistic details and crosstalk between statins and RT in PCa cells remain unknown. In this study, radioresistant DOC-2/DAB2 interactive protein (DAB2IP)-deficient PCa cells were used to evaluate whether simvastatin could enhance the effect of ionizing radiation (IR). The crucial molecules that associated with simvastatin elevated radiosensitivity in PCa cells were explored. Our results demonstrated that a combination treatment with simvastatin and IR synergistically induced apoptosis of radioresistant PCa cells. In addition, simvastatin appeared to compromise DNA double-strand breaks repair by activating the expressions of histone 2A family member X (γ-H2AX) and phospho-checkpoint kinase 1 (p-CHK1), suggesting an underlying mechanism for this radiosensitization of PCa cells. These findings reveal that simvastatin may be a potent therapeutic agent for co-treatment with radiation to overcome radioresistance in PCa cells.

## Introduction

Prostate cancer (PCa) is one of the most prevalent male cancers in western world and the second most frequently diagnosed cancer among males worldwide (Wadajkar et al., 2013). For patients with elevated risk factors or cancer recurrence, radiation therapy (RT) is one of the effective treatment options (Dearnaley et al., 1999; Liu et al., 2014c). However, a significant proportion of patients develop metastatic disease where RT is ineffective due to the development of radioresistance in PCa cells (Hummerich et al., 2006; Liu et al., 2014b).

DOC-2/DAB2 interactive protein (DAB2IP), a member of Ras GTPase-activating protein (GAP) family, functions as a tumor suppressor gene to modulate PCa development (Chen et al., 2002; Tsai et al., 2014). Decreased DAB2IP expression enhances PCa cell proliferation and induces epithelial-mesenchymal transition (EMT), resulting in radioresistant ability (Xie et al., 2009, 2010; Kong et al., 2010). Therefore, there is an urgent need to develop new strategies to overcome radioresistance in PCa cells.

Statins are inhibitors of 3-hydroxy-3-methyl-glutaryl coenzyme A (HMG-CoA) reductase that decrease cholesterol synthesis which are necessary molecules for critical cellular functions (Hebert et al., 1997). The effects of statins in enhancing radiosensitivity in several cancer cell types have been reported (Fritz et al., 2003; Lim et al., 2015). Clinical studies have shown that statins decrease the incidence of advanced PCa, including radioresistant PCa (Hindler et al., 2006; Pisanti et al., 2014). In addition, our recent studies using a population-based cohort demonstrated that statin prescriptions remarkably reduced the risk of death in PCa patients undergoing radiotherapy (Sun et al., 2015; Chen et al., 2018). Although the radiosensitizing properties of statins and their potential effects in inhibiting PCa have been reported, the molecular mechanism underlying this outcome has not been clearly explored (Alfaqih et al., 2017).

Several cell-based studies have suggested that statins exert therapeutic effects on PCa cells, but none of these studies employed the DAB2IP knockdown (KD) (shDAB2IP) PC-3 cell line, a more malignant and radioresistant type of PCa cells (Kong et al., 2010; Wu et al., 2013). In this study, we investigated the cytotoxic effects of simvastatin and the molecular mechanisms of its therapeutic effects on radioresistant PCa cells.

## Materials and Methods

### Antibodies and Reagents

Antibodies against histone-γ-H2AX and phospho-CHK1 were purchased from Cell Signaling (Danvers, MA, United States). Antibodies specific to PARP, Bcl-2, Bax, cleaved caspase 3, were purchased from Santa Cruz Biotechnology (Santa Cruz, CA, United States). All other chemicals or reagents were purchased from Sigma-Aldrich (St. Louis, MO, United States).

### Cell Culture

The methods for the construction of shDAB2IP and control shVector clones, cell transfection, and clone selection were described previously (Xie et al., 2010). The shRNA system (pGIPZ-lentiviral-shRNAmir from Open Biosystems, Huntsville, AL, United States) was used to KD endogenous DAB2IP in prostate epithelial cell line, which was selected by using puromycin. The PC3-KD and PC3-Con (control shVector) cell lines were maintained in Roswell Park Memorial Institute (RPMI) 1640 medium (Hyclone, Logan, UT, United States) supplemented with 5% fetal bovine serum (FBS) (Hyclone). LAPC4-KD and LAPC4-Con (control shVector) cells were maintained in Iscove’s modified Dulbecco’s medium (IMDM) (Gibco, Grand Island, NY, United States) supplemented with 5% FBS. The cells were incubated in a humidified atmosphere containing 5% CO_2_ at 37°C.

### Ionizing Radiation

Cells were irradiated at room temperature in ambient air using the Faxitron RX-650 irradiator (Faxitron X-ray, Wheeling, IL, United States) at the indicated doses which were needed in each experiment.

### Cell Viability Assay

The sulforhodamine B (SRB) assay (Sigma-Aldrich) was used to determine the effects of simvastatin on the growth of PC3 and LAPC4 cells. Cells were treated with various concentrations of simvastatin for different periods. Cells were fixed and stained with SRB pre-mixed solution (0.4% SRB dissolved in 0.1% acetic acid). Cell viability was determined by a spectrophotometer (Bio-Rad, Hercules, CA, United States) at the wavelength of 565 nm.

### Clonogenic Survival Assay

The clonogenic survival assay was used to analyze the surviving fraction (SF) by following our previous study (Lai et al., 2014). PC3-KD cells were seeded for 24 h to allow cell attachment and then treated with increasing doses of IR alone (0–6 Gy), or simvastatin (20 μM) combined with IR. After 7 days’ incubation, the colonies were fixed and stained with 4% formaldehyde in PBS containing 0.05% crystal violet. The number of surviving colonies were counted for evaluation of cell viability. Dose-survival curves for each experiment were constructed by plotting the mean SFs semi-logarithmically as a function of IR dose (Chen et al., 2017). The data were analyzed and survival curves were plotted following the linear quadratic (LQ) model using GraphPad Prism 6.0 software (GraphPad Software, United States).

### Cell Cycle Analysis

PC3-KD cells were treated with simvastatin alone (100 μM), IR alone (2 Gy), or simvastatin combined with IR for 48 h. The treated cells were prepared and stained with 20 μg/ml propidium iodide (Sigma-Aldrich) for 1 h. The stained cells were determined by FACScalibur flow cytometer (Becton-Dickinson, San Jose, CA, United States) and the cell cycle distribution was assessed by using Cell Quest software WinMDI (Verity Software House, Topsham, ME, United States) as described previously (Lin et al., 2017).

### Comet Assay

Comet assay was employed to determine double-strand breaks (DSBs) in irradiated PC3-KD cells by using a Comet Assay kit (Trevigen, Gaithersburg, MD, United States) according to the manufacturer’s instructions. Briefly, cells were mixed with Comet Assay low-melting agarose at a ratio of 1:10 (v/v) and spread evenly on slides. The cells were treated with Comet Assay lysis solution at 4°C for 1 h, submerged in cold electrophoresis buffer and subjected to electrophoresis at 21 V for 30 min. The cells were stained with SYBR Gold. The percentage of DNA tail moment were evaluated with the TriTek Comet ScoreTM software (Version 1.5.2.6; TriTek, Corp., Sumerduck, VA, United States).

### DNA Ladder Analysis

The Apoptotic DNA Ladder Detection Kit (Thermo Fisher Scientific, Camarillo, CA, United States) was used to determine the level of DNA fragmentation of apoptotic cells. Briefly, the treated PC3-KD cells (6 × 10^5^) were taken as starting material for DNA isolation. Cells were washed with PBS and centrifuged at 1,200 rpm before lysis of the cell pellet. Cells were incubated with Enzyme A at 37°C for 10 min and added Enzyme B then incubated at 50°C for 30 min. DNA was isolated by absolute ethanol and then dissolved in DNA suspension buffer. DNA solution was loaded on 1.2% agarose gel for electrophoresis. Ethidium bromide-stained DNA was visualized under UV light and analyzed.

### Western Blot Analysis

PC3-KD cells were treated with simvastatin alone (100 μM), IR alone (2 Gy), or simvastatin combined with IR for 48 h. The treated cells were harvested and cell lysates were prepared. The samples were then resolved by 10% SDS-PAGE and transferred onto polyvinylidene difluoride (PVDF) membranes (Millipore, Billerica, MA, United States). Membranes were probed with primary antibodies and then incubated with horseradish peroxidase (HRP)-conjugated secondary antibody. The proteins of interest were detected using the ECL Western Blotting Detection Reagents (RE Healthcare, Little Chalfont, United Kingdom) and visualized using Image Quant LAS4000 and TL software (GE Healthcare). The signal intensity of each protein was quantified with the UN-SCAN-IT software (Silk Scientific Corporation, Orem, UT, United States).

### Immunofluorescence Staining

PC3-KD cells (2 × 10^5^ cells/well) were seeded on 13-mm glass coverslip in 6-well plates. After treatment, cells were washed by PBS and fixed with 1% paraformaldehyde (Sigma-Aldrich) and permeabilized with 0.1% Triton X-100 for 10 min. The samples were incubated with γ-H2AX antibody. Samples were washed and then incubated with Alexa Fluor 488 conjugated anti-rabbit antibody (Invitrogen, Carlsbad, CA, United States) for 1 h. Nuclei were counterstained with 4′,6-diamidino-2-phenylindole (DAPI) (0.2 μg/ml) for 15 min. The stained cells were then analyzed under a fluorescence microscope (Carl Zeiss, Göttingen, Germany) with a 63× objective (oil immersion, aperture 1.3). All samples were examined in three independent experiments.

### Statistical Analysis

Statistics analysis comparisons of two groups were evaluated by using Student’s *t*-test and showed as mean ± standard deviation. *P* < 0.05 was considered statistically significant. The statistical software was the SPSS program (version 18.0 for windows, SPSS, Inc., Chicago, IL, United States).

## Results

### Simvastatin Inhibited PCa Cell Proliferation

**FIGURE 1:**
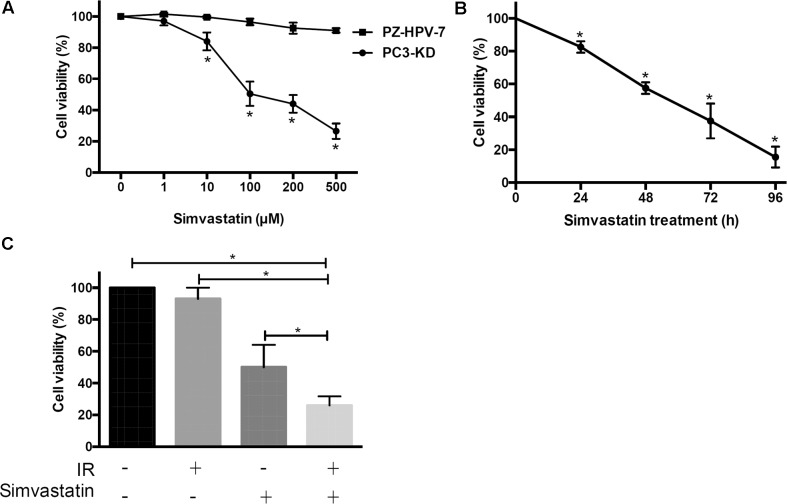
Simvastatin inhibited radioresistant PCa cell proliferation. Cell viability of PZ-HPV-7 and PC3-KD cells in response to simvastatin treatment at various concentrations and durations: **(A)** Simvastatin at various concentrations ranging from 0 to 500 μM was added to cells for 48 h. **(B)** PC3-KD cells were exposed to 100 μM simvastatin for the times indicated (0, 24, 48, 72, and 96 h). **(C)** Cells were treated with ionizing radiation (IR) alone (2 Gy), simvastatin alone (100 μM), or simvastatin combined with IR and were compared to untreated cells. The cell viability was analyzed by sulforhodamine B (SRB) assay. The asterisk (^∗^) indicates statistical significance (*P* < 0.05) as determined by Student’s *t*-test.

To determine the therapeutic potential of statins, we first analyzed whether simvastatin had a cytotoxic effect on radioresistant PCa cells using a DAB2IP KD radioresistant cell lines (Kong et al., 2010). Two DAB2IP-KD clones, PC3-KD and LAPC4-KD, were investigated in this study. Western blot analysis showed that DAB2IP levels were significantly reduced in both DAB2IP-KD lines compared to those in each DAB2IP-control (shVector) cell line (**Supplementary Figure S1A**). In addition, our data showed that simvastatin effectively inhibited cell proliferation in both LAPC4-KD and PC3-KD lines (**Supplementary Figure S1B**). Since simvastatin induced considerably higher apoptosis in PC3-KD cells than that in LAPC4-KD cells, we therefore chose PC3-KD cells as an assay platform for the subsequent experiments. In addition, PZ-HPV-7 cells, an immortalized line derived from benign prostate cells showed sensitizing to radiation (Kong et al., 2015), were used as control group. Cells were cultured with increasing concentrations of simvastatin (0–500 μM) for 48 h, and cell viability was analyzed by SRB assay. Our results indicated that simvastatin hardly showed any effect on the viability of PZ-HPV-7 cells (**Figure 1A**). However, PC3-KD cell proliferation was effectively inhibited by treatment with simvastatin in a concentration- and time-dependent manners (**Figures 1A,B**) with an approximate half-maximal inhibitory concentration (IC_50_) of 100 μM.

Due to the cytotoxic activity of simvastatin on radioresistant PCa cells, we next determined whether simvastatin sensitized PCa cells to radiation. PC3-KD cells were treated with simvastatin alone (100 μM), ionizing radiation (IR) alone (2 Gy), or a combination of simvastatin and IR. Our data showed that survival of PC3-KD cells was significantly decreased after treatment with simvastatin alone or simvastatin plus IR (**Figure 1C**). These results suggest that simvastatin exhibits a significant cytotoxic effect against radioresistant PCa cells.

### Simvastatin Synergistically Sensitized Radioresistant PCa Cells to Radiation

**FIGURE 2:**
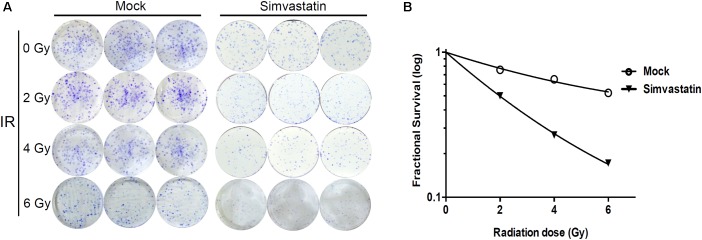
Combined treatment with simvastatin and IR effectively induced cell death. PC3-KD cells were treated with IR alone (0–6 Gy) or simvastatin (20 μM) combined with IR. After incubating for 7 days, cells were **(A)** stained with crystal violet and **(B)** assessed for survival using clonogenic assay as described in Section “Materials and Methods.”

We then examined whether simvastatin could increase susceptibility of radioresistant PCa cells to radiation. PC3-KD cells were treated with IR alone (0–6 Gy) or simvastatin (20 μM) combined with IR, and survival was assessed using a clonogenic survival assay. As shown in **Figure 2**, simvastatin effectively enhanced the radiosensitivity of PC3-KD cells with increasing doses of IR. In addition, the combined treatment with simvastatin and IR significantly increased the susceptibility of cells to IR compared to treatment with IR alone. These results demonstrate that simvastatin sensitizes radioresistant PCa cells to radiation.

### Simvastatin Induced Apoptosis in Radioresistant PCa Cells

**FIGURE 3:**
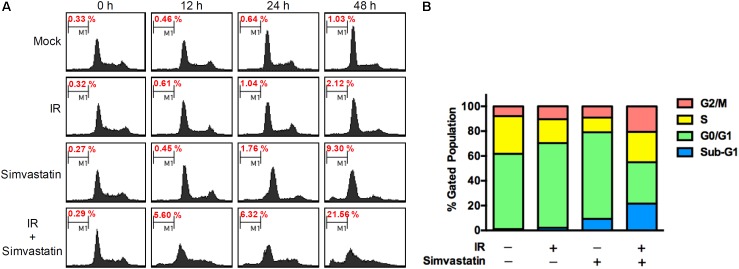
Simvastatin induced apoptosis in radioresistant PCa cells. **(A)** PC3-KD cells were untreated (control), exposed to IR alone (2 Gy), simvastatin alone (100 μM), or simvastatin plus IR, and incubated for 0, 12, 24, and 48 h. Cell cycle distributions based on DNA content was analyzed using flow cytometry. **(B)** The percentages of cells in each phase were calculated.

To further assess whether simvastatin induced apoptosis in PCa cells, PC3-KD cells were treated with simvastatin alone (100 μM), IR alone (2 Gy), or a combination of simvastatin and IR for 0, 12, 24, and 48 h. The distributions of cells in each cell cycle state were determined by flow cytometry. As shown in **Figure 3**, a low percentage of mock and IR-treated cells were in the sub-G1 state from 0 to 48 h. After treating simvastatin for 48 h, the sub-G1 cell population (9.30%) was significantly increased compared to the mock group (1.03%). Notably, there was a dramatic increase in sub-G1 population upon co-treatment with IR and simvastatin following incubation for 48 h (21.56%). These results indicate that simvastatin and IR synergistically enhance cell death in radioresistant PCa cells through apoptosis.

### Co-treatment With Simvastatin and Radiation Increased Biomarkers of DNA Double-Strand Breaks and Apoptosis in Radiosensitive PCa Cells

**FIGURE 4:**
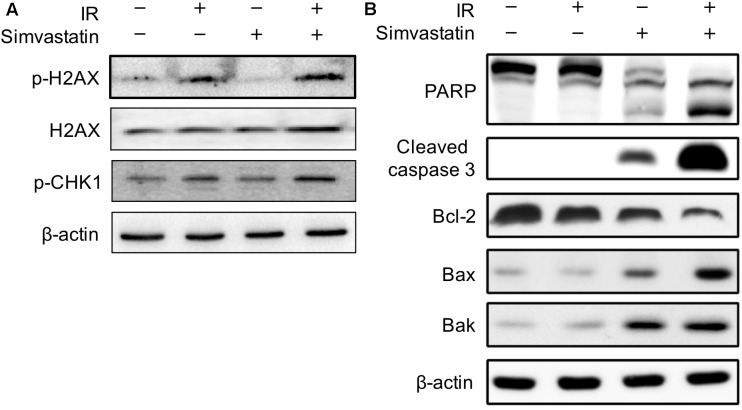
Effects of simvastatin on DSB- and apoptosis-related molecules. PC3-KD cells were treated with IR alone (2 Gy), simvastatin alone (100 μM) or a combination of simvastatin and IR, then incubated for 48 h. Cell lysates were prepared and subjected to western blot analysis using antibodies to probe for the key molecules of **(A)** DSB and **(B)** apoptosis. The data shown are a representative of three independent experiments. β-actin was used as a loading control.

To investigate the molecular mechanism underlying the enhanced IR-induced cell death by simvastatin in PC3-KD cells, the expression levels of key molecules associated with radiation-induced DNA DSBs were investigated. As shown in **Figure 4A**, the expressions of histone 2A family member X (γ-H2AX) and phospho-checkpoint kinase 1 (p-CHK1) were slightly increased in cells treated with IR alone compared to the mock or simvastatin-treated groups. However, the expression levels of these phospho-proteins were remarkably increased following co-treatment with simvastatin and IR. We then analyzed the levels of molecules involved in apoptosis. As shown in **Figure 4B**, there were significant increases in activated PARP and cleaved-caspase 3 in cells co-treated with simvastatin and IR. In addition, simvastatin plus IR treatment effectively inhibited the expression of the anti-apoptotic molecule, Bcl-2, and stimulated the expression of the apoptosis-promoting proteins, Bax and Bak. We then performed DNA ladder assay to examine cell apoptosis. As shown in **Supplementary Figure S2**, mock treatment and IR did not present significant fragmentation. In contrast, cells treated with simvastatin plus IR and incubated for 48 h showed laddering of DNA, which is a characteristic feature of cell apoptosis. These results suggest that simvastatin increases radiation-induced DSB and apoptosis in radioresistant PCa cells.

### Simvastatin-Enhanced Radiosensitivity Was Associated With Compromised DSB Repair

**FIGURE 5:**
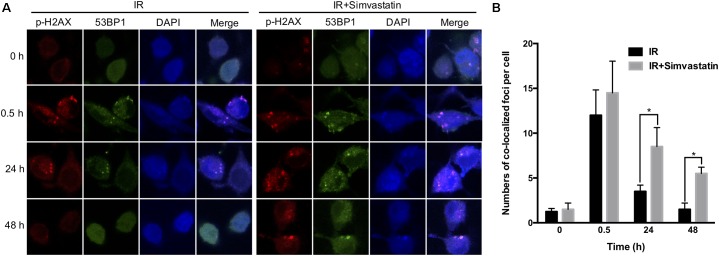
Simvastatin sensitized PCa cells to radiation by compromising DSB repair. PC3-KD cells were treated to IR alone (2 Gy) or co-treated with simvastatin (100 μM) and IR for the indicated times (0, 0.5, 24, and 48 h). **(A)** Cells were immunostained with γ-H2AX (red), 53BP1 (green) and DAPI (blue). **(B)** The colocalization of γ-H2AX with 53BP1 foci in the nuclei were counted for each time point. Statistical significance was evaluated using Student’s *t-*test (^∗^*P* < 0.05).

**FIGURE 6:**
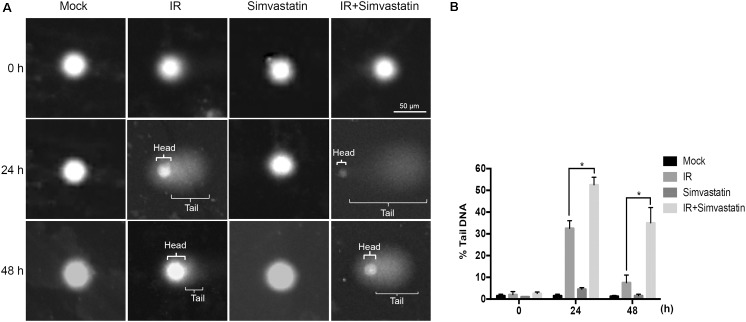
Simvastatin enhances IR-induced DSB in PCa cells. PC3-KD cells were treated to IR alone (2 Gy) or co-treated with simvastatin (100 μM) and IR, then incubation for 0, 24, and 48 h. **(A)** Visualization of the comet assay showing IR-induced DSB in PC3-KD cells **(B)**. The tail moment was quantified for each cell. Statistical significance was evaluated using Student’s *t-*test (^∗^*P* < 0.05).

Recruitment of γ-H2AX and 53BP1 at sites of DNA damage in the nucleus is an early response to DSB (Kong et al., 2010). We therefore examined simvastatin-induced cell death in PCa cells by analyzing foci formation of γ-H2AX and 53BP1. PC3-KD cells were treated with IR (2 Gy) or simvastatin (100 μM) plus IR between 0 and 48 h, and subjected to immunofluorescence staining to detect γ-H2AX and 53BP1 foci. As shown in **Figure 5**, nuclear co-localization of γ-H2AX (red) and 53BP1 (green) foci significantly increased in PC3-KD cells when exposed to IR (2 Gy) for 0.5 h compared to the untreated group (0 h). The foci formation was observed for 48 h in cells co-treated with simvastatin and IR. However, decreased foci levels were observed in PC3-KD cells treated with IR alone for 48 h compared to those co-treated with simvastatin and IR for 48 h. To strengthen our findings that higher levels of γ-H2AX at 24 and 48 h after irradiation shows persistence of DSB, we conducted comet assays. As shown in **Figure 6**, the comets were significantly increased in cells treated with a combination of IR and statin compared with IR treatment alone at 24 and 48 h. Following 48 h incubation, IR only slightly increased the comets. Nevertheless, in cells treated with IR plus simvastatin, high levels of comets remained observable at 48 h. Together, these results demonstrate that simvastatin sustains IR-induced DSB in radioresistant PCa cells.

## Discussion

Radiation therapy is used to damage DNA in PCa cells by IR to increase apoptosis (Chang et al., 2014). However, a certain proportion of patients do not respond to this treatment and become metastatic disease due to intrinsic or the development of radioresistance in PCa cells (Hummerich et al., 2006; Liu et al., 2014a). In this study, we have shown that the co-treatment of simvastatin and IR enhanced susceptibility of PCa cells to IR compared to IR treatment alone. These results demonstrate that simvastatin and IR work synergistically to enhance radiation-induced cell death in radioresistant PCa cells.

Statins that inhibit HMG-CoA reductase, the rate-limiting enzyme in cholesterol biosynthesis, are widely prescribed for lowering serum cholesterol (Hebert et al., 1997). Several preclinical studies indicated that statins prevent proliferation of PCa cells by interfering with these pathways in various ways. In a bone marrow stroma (BMS) model, Brown et al. (2012) showed that statins dramatically reduced prostate epithelial cell invasion toward BMS. The authors suggested that geranylgeranyl pyrophosphate and mevalonate, precursors in cholesterol synthesis, restored the invasive ability of PCa cells treated with simvastatin (Brown et al., 2012). In addition, statins impair the tumor metastasis by inhibiting extracellular matrix degradation, cell migration, and invasion (Hindler et al., 2006). The protective function of stains, included reducing risk of PCa cells that arrest cell cycle, induce apoptosis, leading to autophagy and inhibition of tumor metastasis (Papadopoulos et al., 2011; Zhang et al., 2013). Hoque et al. (2008) reported that apoptosis of PCa cells was induced by lovastatin and simvastatin through both cytochrome *c*-dependent and -independent signaling pathways, involving caspase 3, 8, and 9. Statins may sensitize cells to radiation by arresting cell cycle in the late G1 phase (Chan et al., 2003). Further, our current study focused on the molecular mechanism of statin effects, which effectively enhanced radiation-induced DSB to cause apoptosis. Taken together, these results suggest that statins act as a radiosensitizer and may be used as new therapeutic strategy to treat PCa in combination with RT.

Increased DSB are accompanied by the formation of histone γ-H2AX molecules in the cell nuclei (Bonner et al., 2008). The triggering of checkpoint responses to radiation is one of the major barriers for preventing carcinogenesis (Bartek et al., 2007). In this study, co-treatment with simvastatin and IR highly elevated the expressions of phospho-proteins, including γ-H2AX and p-CHK1. In addition, simvastatin promoted radiation-induced cell death in PCa cells and increased γ-H2AX foci-formation associated with DSB. Our results reveal that the mechanisms underlying radiosensitization induced by simvastatin in PCa cells involves triggering the CHK1 checkpoint response and promotes DSB.

It has been shown that statin was able to counteract radiation-stimulated lung cancer extravasation and metastasis in mice (Hamalukic et al., 2011). The mechanism of statin enhanced radiosensitivity in lung cancer was mediated by inhibiting the PI3K/AKT and activating the tumor suppressor AMP-activated kinase (AMPK) pathway (Sanli et al., 2011). Similar results were obtained by using xenograft mouse models showing that statin sensitized gastric and colorectal cancer cells to radiotherapy through modulation of the ATM/AMPK/p21 pathway (Lim et al., 2015). Our present study showed that the combination therapy using simvastatin and radiation significantly elevated the expression of γ-H2AX and p-CHK1, which enhanced the occurrence of DSB; these observations may be due to the activation of ATM.

A recent study showed conflicting results indicated that lovastatin mediates protection from radiation-induced lung injury (Ziegler et al., 2017). This protection of lung endothelial cells from radiation-induced caspase-dependent apoptosis may involve p53-regulated mechanisms. In contrast, it showed that statin enhanced sensitivity to radiotherapy in lung cancer by inhibiting the p53/p21 signaling pathway (Sanli et al., 2011). A similar study reported that simvastatin synergistically enhanced the effects of radiation in gastric and colorectal cancer cells (Lim et al., 2015). Our present study showed that simvastatin enhanced radiation-induced apoptosis by inhibiting DNA repair in PC3 cells, which were p53-deficient. Therefore, this may explain the cell type-specific diverse effects of statin on IR-induced apoptosis and DSB repair (Ziegler et al., 2017).

Although our study has demonstrated that simvastatin functions as a radiosensitizer and may be used to treat PCa in combination with RT, the limitation of this study is that it lacks *in vivo* data. Accordingly, it is worth to investigate *in vivo*, which will fill the gap in the translational studies.

## Conclusion

Our findings demonstrate that simvastatin can enhance the effects of IR in radioresistant PCa cells. The molecular mechanism of this radiosensitization may be attributed to enhancement of the checkpoint response process and sustained reduction of the DSB repair pathway. This study indicates that simvastatin could be a potential personalized medicine in combination with radiotherapy to target PCa cells, particularly those that are radioresistant.

## Author Contributions

J-TH, C-HTang, and C-HL: conception or design of this work. Y-AC, H-WS, Y-CL, H-YH, C-LW, and C-HTsai: experimental study. Y-AC, H-WS, Y-CL, H-YH, and C-HTsai: data analysis and interpretation. Y-AC, C-HTang, and C-HL: writing the manuscript. All authors provided final approval of the manuscript.

## Conflict of Interest Statement

The authors declare that the research was conducted in the absence of any commercial or financial relationships that could be construed as a potential conflict of interest.

## Supplementary Material

FIGURE S1 Simvastatin increased radio-sensitivity in DAB2IP-knockdown PCa cells. **(A)** Western blot analysis of DAB2IP expression in DAB2IP-control (shVector) and knockdown (shDAB2IP) PCa cells. **(B)** Cell viability of DAB2IP-knockdown cells in response to simvastatin and/or IR was analyzed. Statistical significance was evaluated using Student’s *t-*test (^∗^*P* < 0.05).

FIGURE S2 Analysis of DNA ladder in apoptotic PC3-KD cells. PC3-KD cells were treated to ionizing radiation (IR) alone (2 Gy) or co-treated with simvastatin (100 M) and IR, then incubation for 48 h. DNA was extracted from cells and subjected to agarose gel electrophoresis. M, 1 Kb ladder.
