# Supplementary figures and images for: Simvastatin Sensitizes Radioresistant Prostate Cancer Cells by Compromising DNA Double-Strand Break Repair
Source: Front Pharmacol. 2018 Jun 13;9:600. doi: 10.3389/fphar.2018.00600 (PMC6008406; doi:10.3389/fphar.2018.00600)

Supplementary Figure 1

A

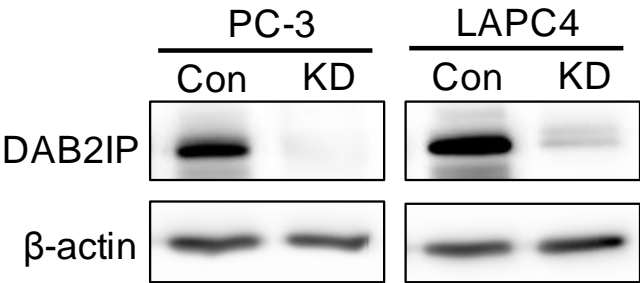

B

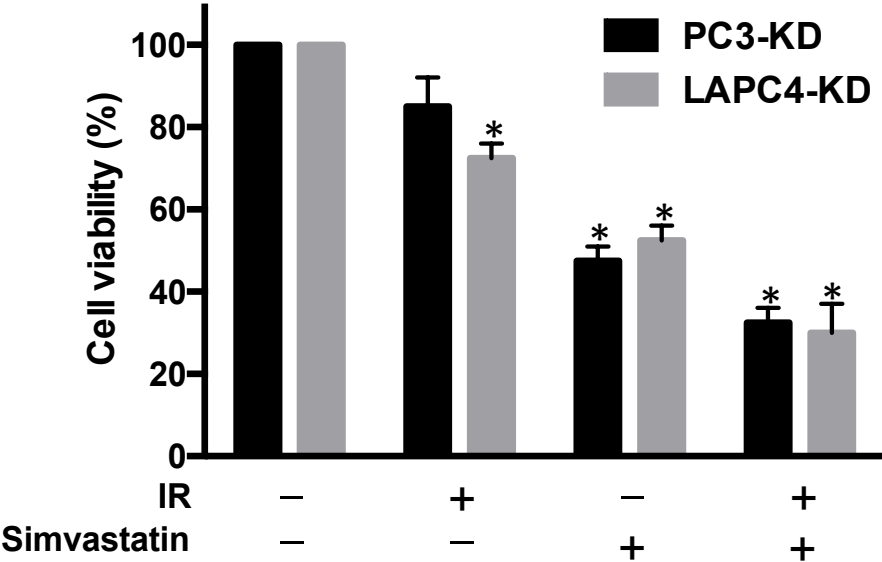

Supplementary Figure 2

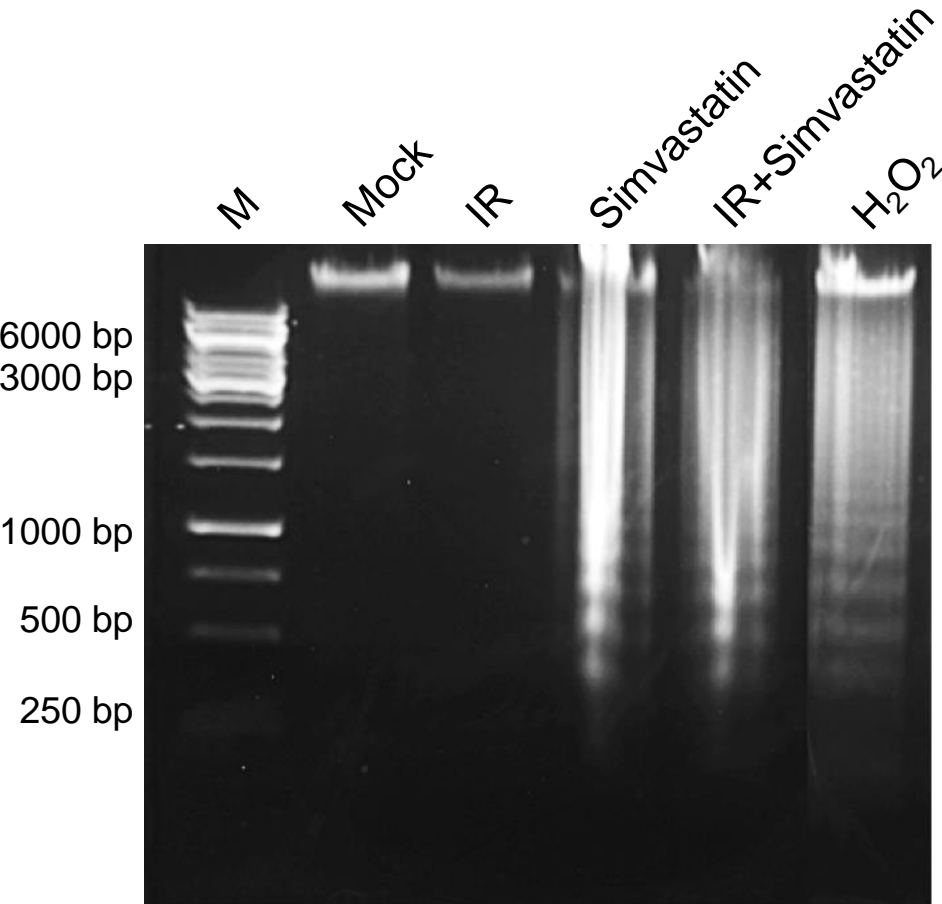

Supplement: FIGURE S1 — Simvastatin increased radio-sensitivity in DAB2IP-knockdown PCa cells. (A) Western blot analysis of DAB2IP expression in DAB2IP-control (shVector) and knockdown (shDAB2IP) PCa cells. (B) Cell viability of DAB2IP-knockdown cells in response to simvastatin and/or IR was analyzed. Statistical significance was evaluated using Student’s t-test (∗P < 0.05). [file Presentation_1.PDF]
